# Combined Use of CFTR Correctors in LGMD2D Myotubes Improves Sarcoglycan Complex Recovery

**DOI:** 10.3390/ijms21051813

**Published:** 2020-03-06

**Authors:** Marcello Carotti, Martina Scano, Irene Fancello, Isabelle Richard, Giovanni Risato, Mona Bensalah, Michela Soardi, Dorianna Sandonà

**Affiliations:** 1 Department of Biomedical Sciences, University of Padova, Via U. Bassi 58/b 35131 Padova, Italy; 2 Généthon INSERM, U951, INTEGRARE Research Unit, Univ Evry, Université Paris-Saclay, 91002 Evry, France; 3 INSERM, Institut de Myologie, U974, Center for Research in Myology, Sorbonne Université, 75013 Paris, France

**Keywords:** sarcoglycanopathy, myogenic cells, myotubes, folding-defective proteins, protein folding correctors, CFTR correctors, proteostasis regulators

## Abstract

Sarcoglycanopathies are rare limb girdle muscular dystrophies, still incurable, even though symptomatic treatments may slow down the disease progression. Most of the disease-causing defects are missense mutations leading to a folding defective protein, promptly removed by the cell’s quality control, even if possibly functional. Recently, we repurposed small molecules screened for cystic fibrosis as potential therapeutics in sarcoglycanopathy. Indeed, cystic fibrosis transmembrane regulator (CFTR) correctors successfully recovered the defective sarcoglycan-complex in vitro. Our aim was to test the combined administration of some CFTR correctors with C17, the most effective on sarcoglycans identified so far, and evaluate the stability of the rescued sarcoglycan-complex. We treated differentiated myogenic cells from both sarcoglycanopathy and healthy donors, evaluating the global rescue and the sarcolemma localization of the mutated protein, by biotinylation assays and western blot analyses. We observed the additive/synergistic action of some compounds, gathering the first ideas on possible mechanism/s of action. Our data also suggest that a defective α-sarcoglycan is competent for assembly into the complex that, if helped in cell traffic, can successfully reach the sarcolemma. In conclusion, our results strengthen the idea that CFTR correctors, acting probably as proteostasis modulators, have the potential to progress as therapeutics for sarcoglycanopathies caused by missense mutations.

## 1. Introduction

LGMD2D (LGMD-R3 according to the new nomenclature [1]) is a rare autosomal recessive disease affecting striated muscle. It belongs to the group of limb girdle muscular dystrophies because of the involvement of the proximal musculature of the shoulders and pelvic girdle [2]. LGMD2D is caused by mutation in the *SGCA* gene coding for α-sarcoglycan (SG) [3,4,5]. This protein, together with β-, γ- and δ-SG, forms the SG complex, a key component of the dystrophin associated protein complex, significantly contributing in preserving sarcolemma from contraction-induced stress. Moreover, a number of direct or indirect regulative roles have been associated to SG-complex [6,7]. LGMD2D, although heterogeneous, is often characterized by early onset and rapid progression, with people affected becoming wheelchair-bound in the adolescence [8]. Presently, no effective therapy is available for LGMD2D as well as for the other three forms of sarcoglycanopathy (LGMD2E, 2C and 2F, due to mutations in *SGCB*, *SGCG* and *SGCD* genes, respectively [9]). Most of the gene defects responsible for the onset of sarcoglycanopathy are missense mutations [10,11,12,13]. In the last few years, the pathogenic mechanism of the forms of sarcoglycanopathy due to this type of genetic defects has been disclosed. It has been observed that many sarcoglycans with an amino acid substitution are unable to properly fold, are recognized by the quality control system of the cells and delivered to a premature degradation [14,15,16,17]. Consequently, the correct assembly, traffic and localization of the SG-complex is impaired, leading to a global reduction in the structural stability of the sarcolemma. An interesting point is the possibility to rescue the defective sarcoglycan as well as the entire SG-complex, by preventing the degradation of the mutant, acting either at the initial [15,16], intermediate [17] or final step [14] of the pathway. On these premises and taking advantage from the tremendous work done on another genetic disease, cystic fibrosis, that shares with sarcoglycanopathy a similar pathogenic mechanism [18], we elaborated a novel strategy of therapeutic intervention [19]. Our approach is based on the use of small molecules known as CFTR correctors, which are effective in rescuing the type II mutants of the cystic fibrosis transmembrane regulator (CFTR) [20,21]. CFTR correctors have been demonstrated effective not only on CFTR mutants but also on structurally correlated [22] as well as structurally uncorrelated defective proteins [23,24]. In our previous paper, incubation of cells, models of sarcoglycanopathy with a number of CFTR correctors, resulted in an increase of the mutated α-SG and the localization at the plasma membrane. Efficacy of one of these small molecules, C17, was subsequently confirmed in myogenic cells from a LGMD2D patient, where we also observed a reduction of the sarcolemma fragility [19]. Here, by using the human pathologic myotubes, we tested the efficacy of additional correctors belonging to the bithiazole family of C17, such as C13 [25], and of the quinoline family, such as C9 and C6 [25,26]. Furthermore, we evaluated two compounds, VX809 and VX661, presently used in combination with the potentiator VX770 for the treatment of CF patients carrying the ΔF508-CFTR mutation [27,28]. The combined administration of C17 with other correctors highlighted the additive and even a potential synergistic activity of such compounds. This foresees the possibility to scale down the dose of the molecules conserving the maximum effect. Moreover, the collected data provided preliminary suggestions concerning the possible mechanism of action. Finally, in the perspective of the development of a cure for sarcoglycanopathy, we also evaluated the ability of the corrected α-SG mutant to interact with the wild type partners as well as the stability at the sarcolemma of the rescued complex.

## 2. Results

### 2.1. Rescue of α-SG by CFTR Correctors in LGMD2D Myotubes

**Figure 1:**
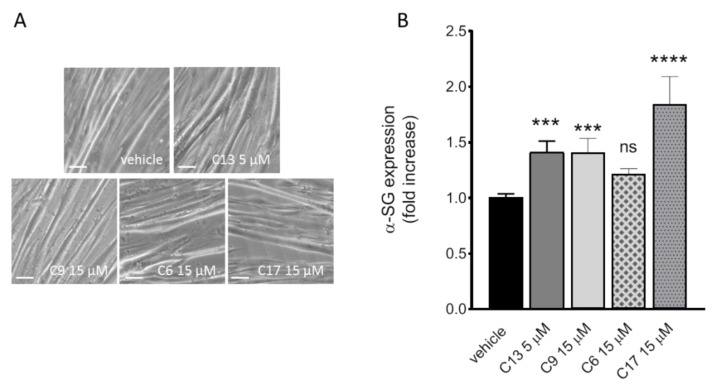
Rescue of α-SG by cystic fibrosis transmembrane regulator (CFTR) correctors in LGMD2D myotubes. Myogenic cells from a patient carrying the L31P/V247M α-SG mutations were differentiated for 7 days and treated for the last 72 h with either 1‰ DMSO (vehicle), C13, C9, C6 or C17 at the indicated concentrations. (**A**) phase contrast images of myotubes at the end of corrector incubation are indistinguishable from the control ones. Scale bar corresponds to 100 µm. (**B**) Densitometric analysis of western blot experiments performed with total protein lysates from LGMD2D myotubes treated as indicated. The α-SG protein was revealed by using specific primary antibody and normalized by the content of β-actin. α-SG content in different samples was expressed as the fold increase (+/− SD) of the amount present in the vehicle treated myotubes, and is the mean value of 3-4 independent experiments (each performed in duplicate). Statistical analysis was performed by One-way ANOVA test followed by multiple comparisons Dunnett’s test; n.s., *p* > 0.05; ***, *p* ≤ 0.001; ****, *p* ≤ 0.0001.

In Carotti et al. [19], we checked the ability of a few number of CFTR correctors in rescuing α-SG mutants in cell models. One of them, corrector C17, was in depth analyzed for its efficacy in myogenic cells from a LGMD2D patient, a compound heterozygote for the L31P and V247M mutations on the *SGCA* alleles. Here, we aim to broaden the number of validated compounds, tested in single or combined administration, potentially relevant for the treatment of sarcoglycanopathy. As model systems, we took advantage of the same pathologic myogenic cells as well as of those of healthy subjects. In both cases, seven-day differentiation of myogenic cells resulted in elongated, multinucleated myotubes as already reported [17,19,29]. We evaluated the following correctors: C13, a compound, like C17, belonging to the bithiazole family [25,30]; C9 and C6 derivative of the quinoline structure [25,26] and C17 used as our reference. Myogenic cells differentiated for seven days, were treated the last 72 h with 1‰ DMSO, used as control vehicle, or with the small molecules at the concentrations reported in Figure 1. The phase contrast images of the LGMD2D myotubes, as appearing at the end of treatment (Figure 1A), show no major alterations in the morphology of the elongated syncytia nor signs of cytotoxicity, such as membrane blebbing or cells detaching from the plate, as expected according to [19]. Figure 1B reports the densitometric analysis of western blot experiments in which the total protein lysates from treated myotubes were probed with specific antibodies for α-SG and β-actin, the latter used to normalize the protein loading. CFTR correctors efficacy was evaluated as the ability to induce an increase of the content of α-SG in comparison to the negative control myotubes (vehicle treated). All the CFTR correctors tested, with the exception of C6, induced approximately 1.5 fold increase of the α-SG content, with C17 being the most effective compound, almost doubling the amount of the protein, in accordance with our previous data [19]. On the contrary, treatments with the same correctors performed on myotubes from a healthy donor elicited no statistically significant effect on the α-SG content (Figure S1). These data confirm what observed in [19], i.e. that CFTR correctors are specifically effective on mutated sarcoglycans.

### 2.2. Additive Effect of C13 in Combination with C17

**Figure 2:**
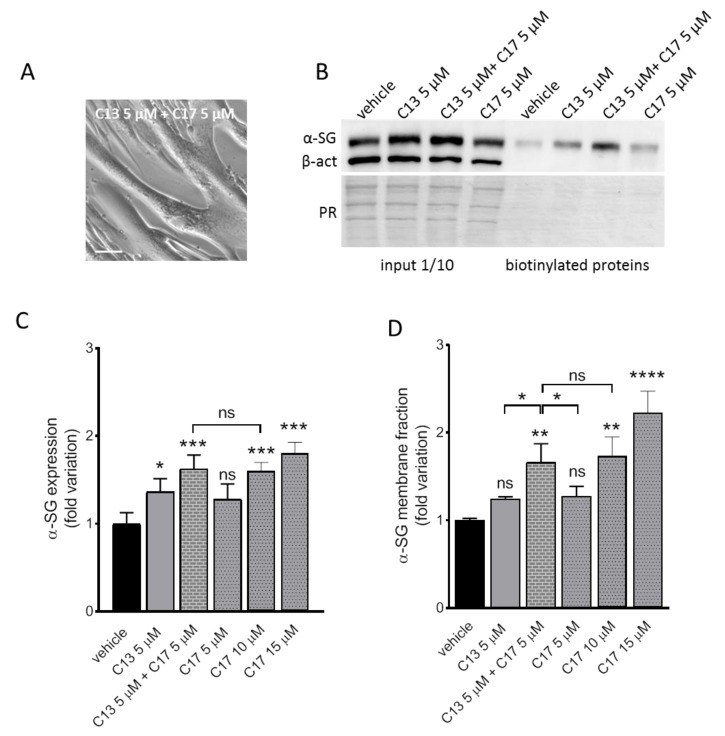
Additive effect of C13 in combination with C17. Myogenic cells from a patient carrying the L31P/V247M α-SG mutations were differentiated for 7 days and treated for the last 72 h with either 1‰ DMSO (vehicle), C13, C17 or C13 + C17 at the indicated concentrations. At the end of incubation, surface proteins were biotinylated. Then, myotubes were lysed and, after quantification, 50 µg of proteins were subjected to pull down assay by streptavidin-conjugated agarose beads. Recovered surface proteins and 1/10 of the starting myotubes lysates (input) were analyzed by SDS-PAGE and western blot with antibodies against α-SG and the cytosolic protein β-actin used as loading control (western blot of input) and to check the absence of biotin internalization (western blot of biotinylated proteins). (**A**) Phase contrast image showing the LGMD2D myotubes at the end of the combined treatment. Scale bar corresponds to 100 µm. (**B**) Representative western blot in which the left part shows the immunodetection of α-SG and β-actin present in the input lysates and the right part the fraction present at the sarcolemma; under the blot, the Ponceau Red staining of the membrane is reported as control of protein loading. Representative western blots of myotubes treated with vehicle, C17 10 µM or 15 µM is reported in Figure S2. (**C**) Quantification of α-SG content by densitometric analysis of the input and **(D)** of the biotinylated fraction of proteins. Values are the mean (+/− SD) of 3–4 independent experiments (each performed in duplicate) and are reported as fold increase of the amount present in the control sample. Statistical analysis was performed by One-way ANOVA test followed by multiple comparisons Tukey’s test; n.s., *p* > 0.05; *, *p* ≤ 0.05; **, *p* ≤ 0.01; ***, *p* ≤ 0.001, ****, *p* ≤ 0.0001.

Subsequently, we evaluated the effect of the combined administration of C17 and C13, both derivatives of the bithiazole moiety [30,31]. We used C17 5 µM, a dose that is ineffective in promoting α-SG rescue in LGMD2D myotubes according to our previous results [19], and C13 5 µM (as in Figure 1), both in single and co-administration. C17 10 and 20 µM was used for comparison. The absence of evident toxic effect elicited by the combined treatment is proved by the phase contrast image of the myotubes, recorded at the end of incubation (Figure 2A). The total α-SG protein content was estimated by western blot and densitometric analysis of myotube lysates. Both C13 and C17 5 µM in single administration promoted a slight increase of the mutated α-SG that was statistically significant for C13 only. On the other hand, the combination C17 + C13 increased the total amount of the mutated α-SG to a level that almost doubled that of the single administrations and is indistinguishable from that elicited by the single administration of C17 10 µM. Finally, as already observed, even a higher increase can be elicited by treating myotubes with C17 15 µM (Figure 2D). To be effective, the rescue must result in the delivery of the recovered protein on the membrane; therefore, we evaluated the fraction of the α-SG mutant present at the sarcolemma by the pull down of biotinylated membrane proteins followed by western blot and densitometric analysis (Figure 2E). It is interesting to note that even if the incubation with C13 5 µM resulted in an increase of the total α-SG mutant content, the fraction able to reach the myotubes surface is considerably lower and not statistically significant. Moreover, in the case of the single administration of C17 5 µM, we can observe a slight increase, not statistically significant, paralleling what observed in the total extract. However, when the combination C13 5 µM + C17 5 µM is used, a large portion of the protein reached the sarcolemma. A similar result was obtained by C17 10 µM. This suggests that these correctors act on the same way, maybe on the same target, according to their structural similarity, improving escape from degradation and traffic of the mutated sarcoglycan toward the final destination.

### 2.3. Additive/Synergistic Effects of C6 in Combination with C17

We tested the combination of low doses of C17 with corrector C9 or C6 both belonging to the class of quinoline derivatives [25,32]. As reported in Figure S3A of supplementary data, C9 in either single or co-administration with C17 resulted in a nearly identical increase of α-SG. On the other hand, this effect did not parallel with an enrichment of the protein at the sarcolemma. Indeed, C9 was ineffective on the traffic of α-SG both when administered alone and in combination with C17 (Figure S3B). Therefore, this corrector was no further investigated.

**Figure 3:**
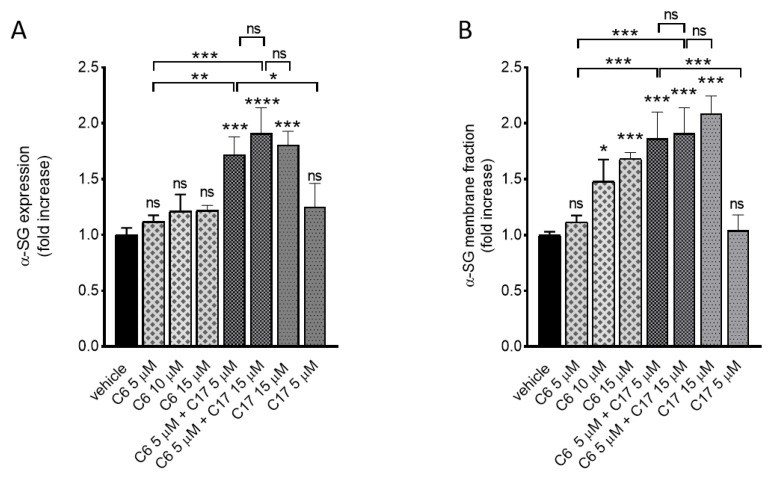
Additive/synergistic effects of C6 in combination with C17. Myogenic cells from a patient carrying the L31P/V247M α-SG mutations were differentiated for 7 days and treated for the last 72 h with either 1‰ DMSO (vehicle), C6, C17 or C6 + C17 at the indicated concentrations. At the end of incubation, myotubes were treated as described in the legend of Figure 2. Representative western blots are reported in Figure S4. (**A**) Quantification of α-SG content by densitometric analysis of the total protein lysates and (**B**) of the biotinylated fraction of proteins. Values are the mean (+/− SD) of 3–4 independent experiments (each performed in duplicate) and are reported as fold increase of the amount present in the control sample. Statistical analysis was performed by One-way ANOVA test followed by multiple comparisons Tukey’s test; n.s., *p* > 0.05; *, *p* ≤ 0.05; **, *p* ≤ 0.01; ***, *p* ≤ 0.001, ****, *p* ≤ 0.0001.

As reported in Figure 3A the incubation of LGMD2D myotubes with corrector C6 at three different concentrations (5, 10 and 15 µM) as well as with C17 5 µM was almost ineffective at the total protein level. In contrast, the incubation with C17 15 µM resulted in a high increase of the α-SG content, as expected. Interestingly, the dual treatment with C6 + C17 both 5 µM, a concentration that was ineffective when compounds were administered alone, produced a robust increase of the α-SG protein, similar to the one obtained with the highest dose of C17. On the other hand, the dual incubation with C6 5 µM + C17 15 µM elicited an effect almost indistinguishable from the one obtained by the sole C17 15 µM (Figure 3A), suggesting that C17 at this dose and in this condition was, by itself, responsible for the maximum effect. An interesting and potentially promising outcome was observed analyzing the α-SG membrane fraction of these samples (Figure 3B). Indeed, with the exception of the lowest concentration of both correctors, a statistical significant increment of the α-SG membrane level was appreciable in all the other conditions. The membrane localization of α-SG was expected in samples in which C17 induced a substantial rescue of the protein (C17 15 µM), as already observed (see for example Figure 2D). The co-administration of C6 + C17 substantially paralleled what observed at the global level. Conversely, the membrane localization of α-SG in cells treated with C6 10 and 15 µM alone (see Figure 3A) suggests that C6 may foster the traffic towards the membrane of the fraction of α-SG that physiologically skips degradation. Remarkably, the effect of C6 became negligible when it was administered in combination with a high concentration of C17, known to induce a significant rescue of the mutant. All this considered, it is possible to suppose an additive/synergistic activity of C6 and C17.

### 2.4. CFTR Correctors Are Effective Only on V247M-α-SG Mutant

**Figure 4:**
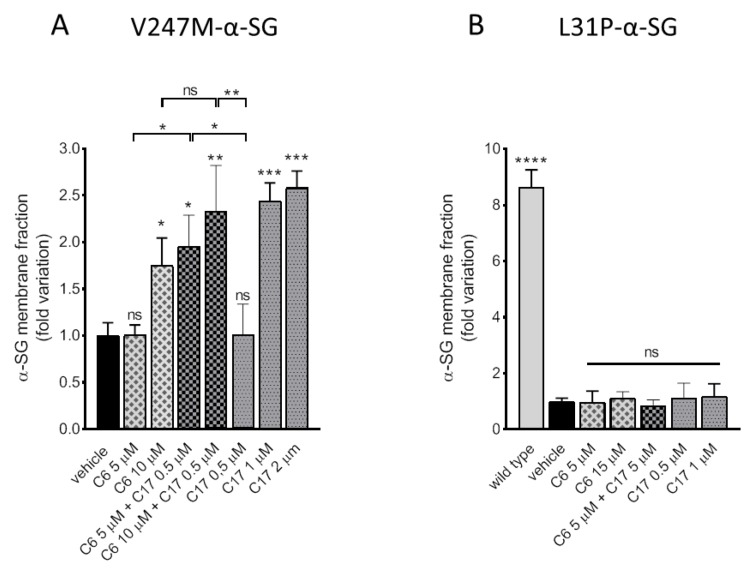
CFTR correctors C6 and C17 are effective on the mutant V247M only. (**A**) HEK293 cells constitutively expressing the V247M mutant of α-SG and (**B**) HEK293 cells transiently expressing the L31P mutant of α-SG were treated for 24 h with either 1‰ DMSO (vehicle), C6, C17 or C6 + C17 at the indicated concentrations. At the end of incubation, cells were lysed and, after quantification, 50 µg of proteins were subjected to pull down assay by streptavidin-conjugated agarose beads. Recovered surface proteins were analyzed by SDS-PAGE and western blot with antibodies against α-SG. Representative western blots are reported in Figure S5. In B, it is reported for comparison the level of a-SG expressed by cells transiently transfected with the wild type form of the protein. Quantification of α-SG content was performed by densitometric analysis. Values are the mean (+/− SD) of 3 independent experiments (each performed in duplicate) and are reported as fold increase of the amount present in the control sample. Statistical analysis was performed by One-way ANOVA test followed by multiple comparisons Tukey’s test; n.s., *p* > 0.05; *, *p* ≤ 0.05; **, *p* ≤ 0.01; ***, *p* ≤ 0.001, ****, *p* ≤ 0.0001.

Considering the genotype of the LGMD2D myotubes used in these experiments, i.e., the presence of different mutations on the two *SGCA* alleles, we cannot exclude that additivity/synergy observed was due to the effect exerted by a single compound on one single mutant. Indeed, our data cannot discriminate if just one or both mutants have been rescued at the sarcolemma. Trying to solve this issue, we treated heterologous cells expressing a single mutant, either V247M-α-SG or L31P-α-SG, with correctors C6 and C17 both in single and co-administration (Figure 4). V247M-α-SG cells are a polyclonal population of HEK293 cells constitutively expressing the mutated form of the human α-SG. As described in [17,19], in these cells, named V-pop, the mutated α-SG moves toward the plasma membrane once recovered by pharmacological treatments. Because of the different sensibility of HEK293 vs myogenic cells towards CFTR correctors, the concentrations here utilized have been selected according to [19]. It is interesting to note that C6 and C17 effectively rescued the mutant and that their activity is presumably synergic. Indeed, the two correctors at a concentration ineffective when use in single administration elicited a strong improvement of α-SG membrane localization when co-administered. The level of V247M-α-SG reached was similar to what obtained by doubling the dose of C17 or C6 and paralleled what observed with LGMD2D myotubes (compare Figure 4A and Figure 3B). A different outcome was obtained when treatments were applied in HEK293 cells transiently transfected with a plasmid expressing the L31P-α-SG mutant. In this case, C6 and C17 were completely ineffective, with no sign of increase of the mutant at the membrane upon either single or combined administration of correctors. As positive control, we evaluated the plasma membrane level of the α-SG protein upon transfection of the wild type form. Altogether these data suggest that the L31P substitution may affect the α-SG structure, making this mutant un-rescuable, at least at these conditions.

### 2.5. Assembly of the Rescued α-SG into the Sarcoglycan Complex 

**Figure 5:**
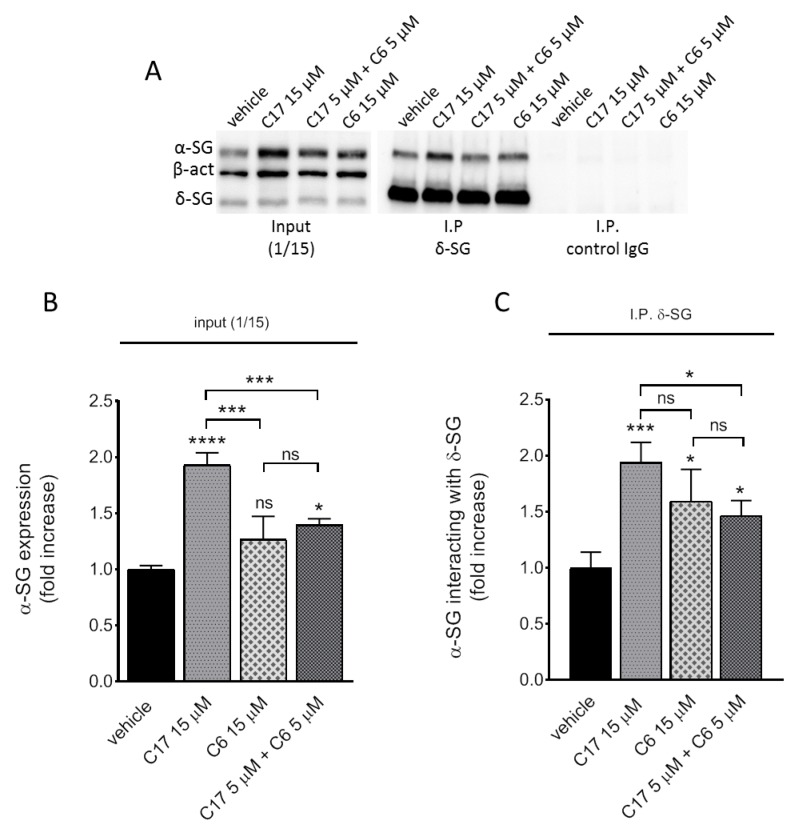
α-SG mutant rescued by correctors C6 and C17 forms a functional SG-complex. Myogenic cells from a patient carrying the L31P/V247M α-SG mutations were differentiated for 7 days and treated for the last 72 h with either 1‰ DMSO (vehicle), C17, C6 or C17 + C6 at the indicated concentrations. At the end of incubation, myotubes were lysed with RIPA buffer without sodium deoxycholate to preserve the sarcoglycans’ interactions. After quantification, 100 µg of proteins were subjected to immunoprecipitation with specific δ-SG mouse monoclonal antibody. As negative control, the same amount of protein from each sample was subjected to immunoprecipitation using an IgG antibody of the same isotype as δ-SG antibody. Immunocomplexes were resolved by SDS-PAGE and analyzed by western blot using the antibodies specific for α-SG, δ-SG and β-actin. (**A**) Representative western blot showing total protein lysates (input) (left part); the immunocomplexes recovered by δ-SG antibody (I.P. δ-SG) (central part); the negative control of immunoprecipitation (I.P. control IgG) (right part). (**B**) Quantification of α-SG content by densitometric analysis of the total protein lysates, normalization for protein loading was performed on the signal of β-actin. (**C**) Quantification of α-SG content by densitometric analysis of the immunocomplexes; normalization was performed on the signal of δ-SG. Values are the mean (+/− SD) of 3 independent experiments (each performed in duplicate) and are expressed as fold increase of the control. Statistical analysis was performed by One-way ANOVA test followed by multiple comparisons Tukey’s test; n.s., *p* > 0.05; *, *p* ≤ 0.05; ***, *p* ≤ 0.001, ****, *p* ≤ 0.0001.

To analyze in pathologic myotubes the ability of the rescued α-SG mutant to interact with the wild type subunits of the sarcoglycan complex, we performed a co-immunoprecipitation assay by using the antibody against δ-SG or β-SG for immunoprecipitation (IP) and the antibodies specific for α-SG and δ-SG for the western blot analysis. It is known that δ-SG and β-SG form a tight core, to which first binds γ-SG and later interacts α-SG, as the last subunit added during tetramer maturation and traffic towards the plasma membrane [33,34,35]. Therefore, the identification of α-SG in the immuno-complexes precipitated by either δ-SG or β-SG antibodies means the presence of a complete, fully functional SG-complex. It is interesting to note in Figure 5C, that the immune-complexes contained a fraction of α-SG that paralleled the rate of rescue elicited by C17 alone or by the dual incubation (as reported in Figure 5B). Remarkably, in myotubes treated with C6 15 µM, the amount of α-SG involved in hetero-tetramer assembly was significantly higher in comparison to untreated cells, even if the accumulation of the protein was negligible (compare Figure 5C with Figure 5B). Superimposable results were obtained when immunoprecipitation was carried out with β-SG antibodies (Figure S5). These data support the idea that C6 acts mainly promoting tetramer formation and consequently traffic towards the sarcolemma.

### 2.6. The Rescued Protein Is Stable at the Plasma Membrane

**Figure 6:**
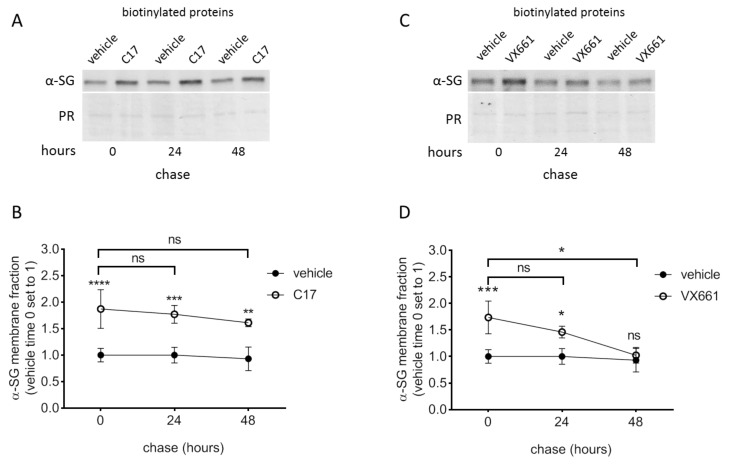
The rescued α-SG protein is stable at the plasma membrane. Myogenic cells from a patient carrying the L31P/V247M α-SG mutations were differentiated for 7 days and treated for the last 78 h with either 1‰ DMSO (vehicle), C17 15 µM or VX661 25 µM. At the end of treatment, corrector-containing medium was removed and replaced with fresh medium. Myotubes were then treated and lysed as described in the legend of Figure 2 at the indicated time points. Representative western blots of the biotinylated membrane fraction from myotubes treated with vehicle or C17 (**A**); with vehicle or VX661 (**C**) probed with antibodies specific for α-SG, Ponceau Red (PR) staining is reported to estimate protein loading. The content of α-SG resident in the sarcolemma of cells pre-treated with C17 (**B**) or VX661 (**D**) was quantified by densitometric analysis of 4 independent experiments. Mean values (+/− SD) are referred to the amount of α-SG present in vehicle-treated cells at time 0. Statistical analysis was performed by One-way ANOVA test followed by multiple comparisons Tukey’s test; ns, *p* > 0.05; *, *p* ≤ 0.05; **, *p* ≤ 0.01; ***, *p* ≤ 0.001; ****, *p*≤ 0.0001.

An important issue to consider in the development of a new therapy is the duration of the effect elicited by the pharmacological treatment. Starting to dissect this point, we treated LGMD2D myotubes with corrector C17 15 µM or VX661 25 µM for 72 h. Then, after correctors withdrawal, myotubes were analyzed 24 and 48 h later to measure both the total α-SG protein content (Figure S10) and, importantly, the fraction present at the sarcolemma (Figure 6). Firstly, it is seen that the amount of the steady state level of α-SG, in cells treated with vehicle, was constant through to the experiments. Conversely, the amount of sarcolemma-resident α-SG, upon corrector withdrawal, diminished during time, because part of the recovered protein is probably recycled from the membrane. However, the membrane removal of the α-SG rescued by C17 was slow, as 88 and 70% of the initial amount of α-SG was still present at the sarcolemma after 24 and 48 h of corrector withdrawal. Moreover, these differences were statistically irrelevant in comparison to the initial amount of the protein (Figure 6A,B). Conversely, the α-SG rescued by VX661 was faster recycled from the membrane, as the level of the protein became not statistically different from untreated samples after 48 h (Figure 6C,D). No statistically significant differences were observed in the level of the δ-SG present at the sarcolemma nor at the end of correctors incubation, neither at the two time points analyzed upon small molecules withdrawal (Figure S11). On the bases of these data, it is possible to suppose that the corrected α-SG mutant, once at the sarcolemma, is quite stable.

## 3. Discussion

Sarcoglycans localize at the plasma membrane of striated muscle. Thus, they belong to that part of the cell proteome translocating into the endoplasmic reticulum (ER) for maturation and eventually entering the secretory pathway to reach the cell surface [9]. In this compartment, a stringent quality control system scrutinizes the newly synthetized proteins in order to: (i) allow the properly folded polypeptides to pass forward and (ii) deliver to degradation those folding-defective [36,37]. This ensures protein homeostasis avoiding, at the same time, the accumulation of potentially harmful species. However, the disposal of mutated proteins failing to attain their native conformation, as in CF or sarcoglycanopathy, may result in a loss of function condition [31]. In the last years, many efforts have been devoted to develop pharmacological strategies aiming at restoring proteostasis in these diseases such as protein replacement and pharmacologic chaperone/kinetic stabilizer approaches, as well as readapting the native biology of the cell by proteostasis regulators [38,39]. In this context, the small molecules screened and developed to recover mutants of the CFTR represent the paradigm of how pharmacological chaperons or proteostasis regulators can be successfully used to treat a severe conformational disease like CF [21,38,40]. We recently established the proof of concept that protein-folding correctors belonging to the family of CFTR correctors are effective in rescuing folding-defective α-SG mutants exogenously expressed in cell models. One of such compounds, corrector C17, was then tested in pathologic myogenic cells from a LGMD2D patient showing the recovery of the SG-complex at the sarcolemma with the concomitant reduction of the membrane fragility [19]. Here, we show evidence about the efficacy of other correctors used in single or combined administration, broadening the number of compounds validated *in vitro*, potentially useful in sarcoglycanopathy and highlighting possible additive/synergic effects for some of them. Furthermore, we observed in heterologous cells that the efficacy of such correctors is mutation dependent, as only the V247M mutant is effectively rescued. Finally, by assessing the stability of the SG-complex upon CFTR correctors’ incubation, we give a first estimation of the beneficial duration of the treatments.

To evaluate correctors’ efficacy, we measured both the global increase of α-SG content but particularly the rise occurring at the sarcolemma, as sign of trafficking rescue of the mutant. CFTR correctors C13, C9 or C17, the latter used as our internal reference, selectively elicited the accumulation of α-SG in pathologic, LGMD2D, myotubes while were ineffective in healthy ones. This supports the idea that these compounds are effective only if a defective sarcoglycan is present, as already observed in [19]. On the other hand, corrector C6 is unable to increase the content of the mutated α-SG in both healthy and LGMD2D myotubes at all the concentrations tested. However, if focusing on the membrane fraction, it is possible to observe that C6 10 or 15 µM increased the localization of α-SG at the sarcolemma (Figure 3B). Membrane localization also occurred with C17 15 µM (Figure 3B), as expected in accordance with [19], on the other hand a strong increase of the total α-SG content was also observed with this concentration of C17. We already evidenced that this corrector is able to increase the amount and stabilize the α-SG mutant expressed by LGMD2D myotubes without affecting its transcription rate [19]. Interestingly, in the case of C6 the significant rise of α-SG at the sarcolemma occurred in the absence of an evident accumulation of the protein. Thus, we may exclude a transcriptional or translational upregulation of α-SG elicited by the corrector. Conversely, this outcome suggests a major role of C6 in promoting the traffic of the portion of mutant that physiologically skips degradation. In support of this view, there are also the IP assays, showing an increased amount of functional hetero-tetramer recoverable from C6-treated myotubes. In this respect, it is important to remind that condition for proper sarcolemma localization is the assembly of the SG-complex [14,41]. The fact that C6 did not improve the membrane localization of α-SG in healthy myotubes may be explained by the saturation of the assembly/transport systems, already working at the maximum rate when the wild type form of each sarcoglycan is present.

As pharmacological chaperons, correctors have been subcategorized in class-I or II depending on the preferential target site on the mutated CFTR protein [42]. Furthermore, there is evidence that the co-administration of CFTR correctors belonging to different classes is beneficial for the rescue at the plasma membrane of several CFTR mutants, enhancing the folding and stability of the channel, thus improving the release from proteostasis components responsible for retention/delivery to degradation [43,44,45]. However, sarcoglycans and CFTR are quite different in terms of both structure and function [45,46]; moreover, the examples of CFTR correctors’ efficacy on proteins different from the chloride channel are growing [23,24,47]. In consideration of this variety of actions, it is likely that, in the absence of the specific target (CFTR) but in the presence of a protein that being mutated affects the proteostasis network, correctors act mainly modulating the function, composition or concentration of elements of the network itself. Consequently, the perspective that different correctors may act on different targets, offers the opportunity to develop combined treatments also for sarcoglycanopathy. We observed that the protein accumulated by either C13 or C17 alone moved toward the membrane, even though the amount successfully translocated was not statistically significant. Conversely, the combination C17 + C13 resulted in the successful rescue of α-SG at the sarcolemma (Figure 2). All this considered, we might hypothesize an additive effect of the two bithiazole derivatives (C17 and C13), acting on the maturation pathway, probably on the same target. To support this view, there is also the outcome of cells treated by doubling the concentration of C17 (10 µM). Indeed, the amount of α-SG mutant gaining the sarcolemma was superimposable to the one obtained by the co-administration of the two small molecules (each one 5 µM). Conversely, the combination C17+C9 had no additional effect in comparison to the single administration (Figure S2). In this case however, neither the single nor the double administration promoted the membrane translocation. The α-SG mutant accumulated by C9, which is a quinoline derivative, probably acquired a conformation not appropriate for assembly and/or traffic, and not even the presence of corrector C17 can overcome the problem.

A different outcome was observed when C6, another quinoline derivative, was administered concurrently with C17 (each one 5 µM). In this case we measured a robust increase of the α-SG sarcolemma localization, paralleling the accumulation of the mutant (Figure 3). This rescue was not statistically different from the one resulting by the activity of the sole C17 15 µM (three times the dose used in co-administration). Similar results were collected also using heterologous cells expressing the V247M-α-SG. This suggests an additive/synergistic effect of the two small molecules, with C17 promoting folding or reducing degradation of the mutant, and C6 improving assembly/traffic towards the sarcolemma, as suggested by the outcome of the single administration. This is an important hint for drug development, as the co-administration may allow scaling down the concentration of the compounds utilized, thus reducing possible side effects. Remarkably, when administered in combination with a high dose of C17 15 µM, there is no additional effect by the presence of C6. If we suppose C6 is mainly involved in the traffic, it is possible that the amount of α-SG accumulated by the activity of C17 15 µM might saturate the traffic flow of the SG-complex. 

Many efforts are presently devoted to the repurposing of available drugs for the treatment of neglected diseases. In this frame, we tested two CFTR correctors, VX661 (Tezacaftor) and VX809 (Lumacaftor) presently on the market in formulation with the potentiator VX770 (Ivacaftor) for CF patients bearing the ΔF508 mutation of CFTR [28,48]. Efficacy of VX661 and VX809 was evaluated in myotubes from both LGMD2D and healthy subjects (Figures S6–S9). Even though modest, the α-SG rescue was evident (Figure S7), especially when VX661 was co-administered with C17 (Figure S9), suggesting possible additive effects of the two small molecules and opening the way for planning novel experiments to identify the useful dose, the timing of incubation, the most effective ratio of the two compounds also aiming to avoid any possible side effect.

Essential issue in drug development is the duration of the effect elicited by the treatment. Our evidence is that, once at the sarcolemma, the SG-complex even if containing a mutated subunit is quite stable. Indeed, 48 h upon C17 corrector withdrawal, the amount of α-SG resident in the sarcolemma was still similar (no statistically different) to that present at the end of the treatment (Figure 6A,B). Even though these experiments need an *in vivo* validation, this is a first indication that the rescue is effective and durable. By our results with LGMD2D myotubes, we cannot know if just one or both the mutated α-SG polypeptides (carrying either the L31P or the V247M amino acid substitution) have been rescued by the treatments. Though, data collected with heterologous cells expressing a single mutant suggest that only V247M-α-SG is rescuable. On the other hand, the second mutation introduces a proline in place of a leucine, close (7 aa apart) to the site of cleavage of the α-SG signal peptide [6]. Thus, even though the CLC bio Workbench software used in [16] predicted no modification on the secondary structure, our data suggest that L31P substitution may have an impact on the structure not easily recoverable. Additional experiments will permit an in-depth investigation of this point, also considering a possible action of correctors as pharmacological chaperone, thus able to bind selectively specific mutants. Anyway, this information may be helpful to foresee the outcome of corrector treatments in relation to the type of mutation. 

In conclusion, our data support the view that several CFTR correctors, probably acting as proteostasis regulators, could be effective in diseases different from CF. Among the molecules tested, corrector C17 may be classified as the most effective in sarcoglycanopathy. On the other hand, the presence of an additional corrector, such as C6, may guarantee a similar effect, reducing concomitantly the dose of the compounds utilized. This means that these small molecules are probably acting on different steps of the same pathway, in either additive or synergistic way. Finally, the α-SG mutant, especially when “corrected” by C17, is stable once inserted in the complex at the sarcolemma. Although preliminary, these data will be of crucial support in the design of the preclinical experiments *in vivo*. Finally, even if a deeper investigation on the mechanism of action of CFTR correctors is mandatory, our findings represent a first crucial step towards the development of a remedy for most of the sarcoglycanopathy patients.

## 4. Material and Methods

### 4.1. Site Directed Mutagenesis

The full-length cDNA encoding human α-sarcoglycan cloned in the pcDNA3 mammalian expression vector was previously described [49]. The L31P missense mutation was introduced with the GeneArt Site Directed Mutagenesis system (Thermo Fisher Scientific, Waltham, MA, USA) according to manufacturer’s instructions, by using the following primer pair: forward 5′-GACCACGCTACACCCACCTGTGGGCCGTGTCTTTG-3′, reverse 5′-CAAAGACACGGCCCACAGGTGGGTGTAGCGTGGTC-3′.

### 4.2. Chemicals, Cell Culture and Treatments

C6, C9, C13 and C17 were from Exclusive Chemistry (Obninsk, Russia) VX809 and VX661 were from Cayman Chemicals (Ann Arbor, MI, USA). All compounds were dissolved in DMSO at 1000× concentration to have the same content of vehicle (1‰) after the dilution at the final concentration used for treatment.

HEK293 (ATCC, Manassas, VA, USA) and V247M cells (a population of HEK293 cells stably expressing the V247M α-SG, described in [17]) were grown in DMEM (Thermo Fisher Scientific, Waltham, MA, USA) supplemented with 10% FBS (Thermo Fisher Scientific, Waltham, MA, USA). For transient expression, cells were seeded at 50,000 cells/cm^2^ on poly-D-Lysine coated plates and transfected the day after seeding with TransIT293 (Mirus Bio, Madison, WI, USA) according to manufacturer’s instruction. Twenty-four hours after transfection, medium was replaced with DMEM supplemented with 2% FBS containing the desired concentrations of correctors dissolved in DMSO. After twenty-four hours of treatment, the membrane localization of α-SG mutants was analyzed by the biotinylation assay.

Immortalized human myoblasts (AB678) were from the ‘Human cell culture platform’ of the Myology Institute in Paris [50]. Primary human myogenic cells from an LGMD2D patient were isolated from a bioptic fragment from the Telethon Genetic Bio-Bank facility (Padova, Italy) [17]. Myogenic cells were grown in Dulbecco’s Modified Eagle Medium (DMEM, Sigma-Aldrich, St. Louis, MO, USA) pH 7.2 supplemented with 30% Foetal Bovine Serum (FBS, Thermo Fisher Scientific, Waltham, MA, USA), insulin 10 μg/mL, Fibroblast Growth Factor (FGF, 25 ng/μL) and Epidermal Growth Factor (EGF, 10 ng/μL) (Sigma-Aldrich, St. Louis, MO, USA). To start differentiation, myoblasts, grown at confluence, were incubated with DMEM supplemented with 2% Horse Serum (Euroclone, Milano, Italy), 10 µg/mL human recombinant insulin (Sigma, St. Louis, MO, USA), 100 µg/mL human transferrin (Sigma-Aldrich, St. Louis, MO, USA). Differentiation was carried out for seven days. CFTR correctors dissolved in DMSO were added 72 h before myotubes lysis or biotinylation. After treatments, cells were washed twice with ice cold PBS and lysed with RIPA buffer (Tris-HCl 50 mM pH 7.5, NaCl 150 mM, NP-40 1% *v*/*v*, sodium deoxycholate 0.5% *w*/*v*, SDS 0.1% *w*/*v*) supplemented with complete protease inhibitor (Sigma-Aldrich, St. Louis, MO, USA).

### 4.3. Biotinylation of Surface Proteins

For the biotinylation reaction, at the end of the experiments, myotubes were incubated at 4 °C for at least 10 min and all the procedures were performed at this temperature to slow down cellular processes and to prevent the internalization of biotin by endocytosis. Cells were then washed three times with ice cold PBS supplemented with calcium and magnesium and incubated under gentle agitation with a solution of 0.5 mg/mL biotin (EZ-Link Sulfo-NHS-LC-Biotin, Thermo Fisher Scientific, Waltham, MA, USA) in PBS for 20 min at 4 °C. The biotinylation reaction was stopped washing the cells twice with 100 mM glycine in PBS (each wash 5 min), and twice with PBS. Cells were lysed with RIPA buffer, and lysates were centrifuged 30′ at 20,000× *g* at 4 °C. The supernatants were quantified by BCA assay, and 50 µg of proteins were incubated with streptavidin agarose resin (Thermo Fisher Scientific, Waltham, MA, USA) (30 µL of resin for each sample) over night at 4 °C. The streptavidin-resin, after the incubation under gentle rotation with myotubes lysates, was washed three times with RIPA buffer. Bound proteins were eluted with Laemmli sample buffer and analyzed by western blot. As negative control, lysate of non-biotinylated cells was incubated with the streptavidin resin and analyzed by western blot to exclude nonspecific binding to the streptavidin resin. The absence of biotin internalization was assessed probing the western blot membranes with an antibody specific for the cytosolic protein β-actin.

### 4.4. Co-Immunoprecipitation

After 72 h treatment, AB678 or 6813 cells were lysed with RIPA buffer without sodium deoxycholate to preserve the interaction between the sarcoglycans and then lysates were centrifuged at 20,000× *g* for 30′ at 4 °C. Protein concentration was determined by BCA assay, and 100 µg of protein was incubated under gentle rotation with 1 µg of anti-delta-SG mouse monoclonal antibody (Leica Biosystem, Wetzlar Germany) or with 1 µg of mouse IgG (Sigma-Aldrich, St. Louis, MO, USA) as negative control, for 2 h at 4°C. Finally, 40 µL of Pure Proteome Protein G magnetic beads (Merck-Millipore, Darmstadt, Germany) were added and after 1 h at 4 °C under gentle rotation, magnetic beads were collected with a magnetic stand and washed three times with RIPA buffer without sodium deoxycholate. Bound proteins were eluted with Laemmli buffer and analyzed by western blot. 

### 4.5. Western Blot Analysis

Proteins were resolved by SDS-PAGE, blotted onto a nitrocellulose membrane and probed with selected antibodies (see below). Secondary antibodies were horseradish peroxidase-conjugated, and blots were developed with ECL chemiluminescent substrate (Euroclone, Milano, Italy), and chemiluminescent signals were digitally acquired with Alliance Mini HD9 Imaging System (Uvitec, Cambridge, UK.). Densitometry was performed with the ImageJ software. The intensities of sarcoglycan bands were normalized for the intensity of the total protein loading, evaluated by Ponceau Red staining of the membranes or β-actin.

### 4.6. Antibodies

Rabbit monoclonal anti α-SG (AB189254) was from Abcam (Cambridge, UK); mouse monoclonal antibodies specific for β-SG, δ-SG and γ-SG were from Leica Biosystem (Wetzlar Germany) mouse monoclonal anti β-actin was from Sigma-Aldrich (St. Louis, MO, USA). Rabbit polyclonal antibody specific for α- and δ-SG were produced as previously described [17]. Secondary antibodies were horseradish peroxidase-conjugated goat anti-mouse or anti-rabbit IgG (Sigma-Aldrich, St. Louis, MO, USA).

### 4.7. Statistical Analysis

Values are expressed as means ± SD. Before performing the statistical analysis, data were checked for normal distribution with the Kolmogorov–Smirnov normality test (GraphPad Software, San Diego, CA). Statistical differences among groups were then determined by One-way ANOVA test, followed by either Dunnett’s test for simultaneous multiple comparisons with control, or Tukey’s test for simultaneous comparisons of all possible contrasts (pairs). A level of confidence of *p*  <  0.05 was used for statistical significance.

## Supplementary Materials

Supplementary materials can be found at https://www.mdpi.com/1422-0067/21/5/1813/s1.
