# Supplementary material for: Combined Use of CFTR Correctors in LGMD2D Myotubes Improves Sarcoglycan Complex Recovery
Source: Int J Mol Sci. 2020 Mar 6;21(5):1813. doi: 10.3390/ijms21051813 (PMC7084537; doi:10.3390/ijms21051813)
Supplement: Supplementary file 1 [file ijms-21-01813-s001.pdf]

S1

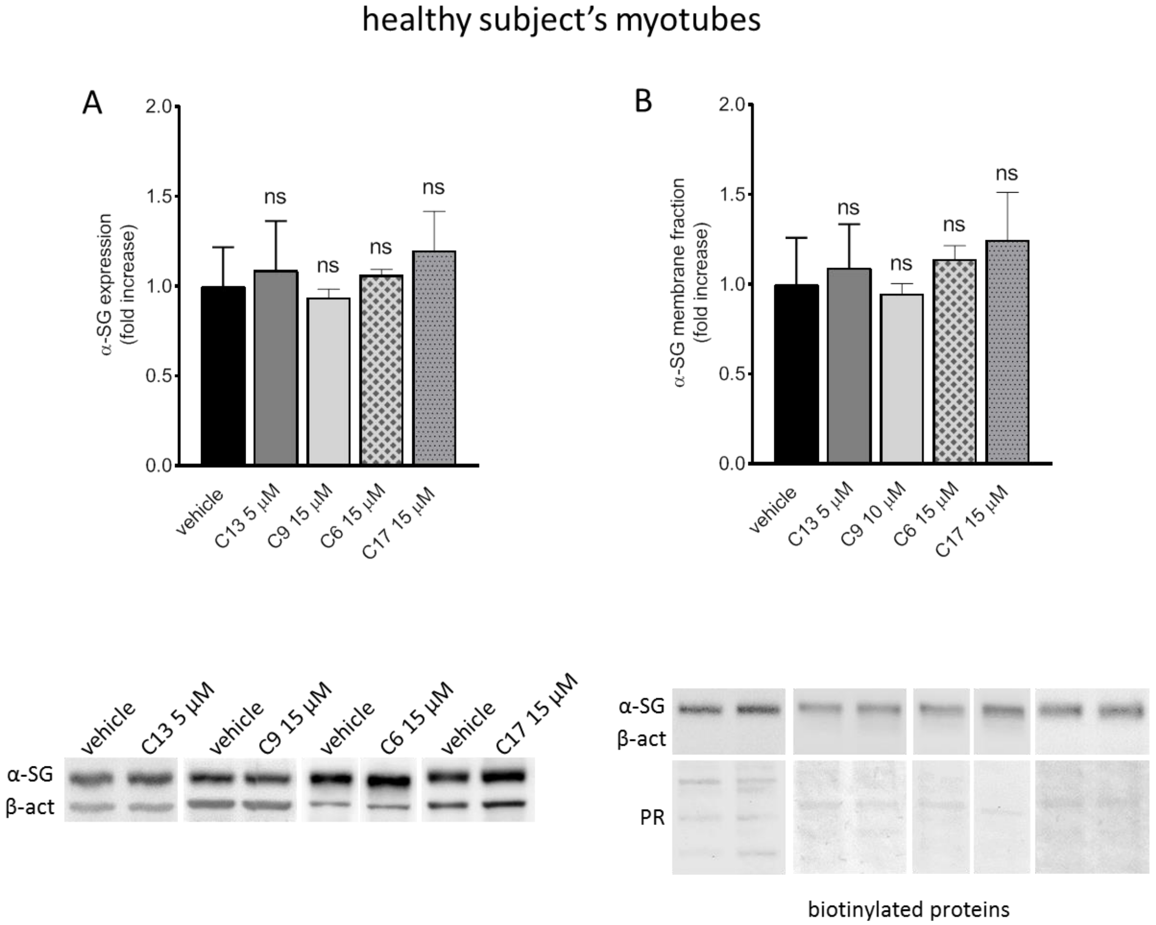

S1: CFTR correctors are ineffective in healthy subject's myotubes

Myogenic cells from a healthy subject were differentiated for 7 days and treated for the last 72 h with either 1‰ DMSO (vehicle), C13 5 µM, C9 15 µM, C6 15 µM or C17 15µM. At the end of incubation, myotubes were either lysed and total proteins analysed by western blot or subjected to biotinylation. After lysis, 50 µg of myotubes biotinylated proteins were subjected to pull down assay by streptavidin-conjugated agarose beads. Surface protein were subsequently analysed by western blot. **(A)** Quantification of α-SG content in myotube lysates was performed by densitometric analysis of 4 independent western blot experiments. Representative examples are reported below the graph, α-SG protein was revealed with a specific primary antibody; β-actin staining was utilized to normalize the total amount of proteins loaded in each lane. No statistical difference was observed among CFTR correctors and vehicle treated samples. **(B)** Quantification of the sarcolemma fraction of α-SG was performed by densitometric analysis of 4 independent western blot experiments. Representative examples are reported below the graph, α-SG protein was revealed with a specific primary antibody; β-actin staining was utilized to check the absence of biotin internalization, Ponceau Red is reported to estimate protein loading. No statistical difference was observed in the content of the α-SG present at the sarcolemma among different samples. Statistical analysis was performed by One-way ANOVA.

S2

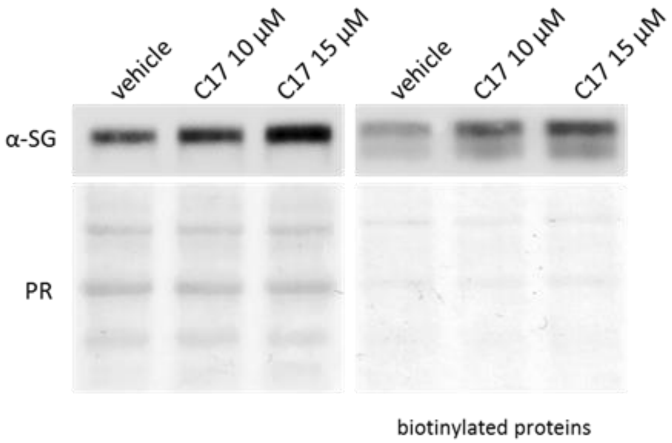

S2: Effects of C17 on LGMD2D myotubes (representative Western blots).

Myogenic cells from a patient carrying the L31P/V247M α-SG mutations were differentiated for 7 days and treated for the last 72 h with either 1‰ DMSO (vehicle), C17 at the indicated concentrations. At the end of incubation, myotubes were either lysed and total proteins analysed by western blot or subjected to biotinylation as described in the legend of S1.
α-SG protein was revealed with a specific primary antibody; Ponceau Red (PR) staining of the membranes was reported as control of protein loading.

S3

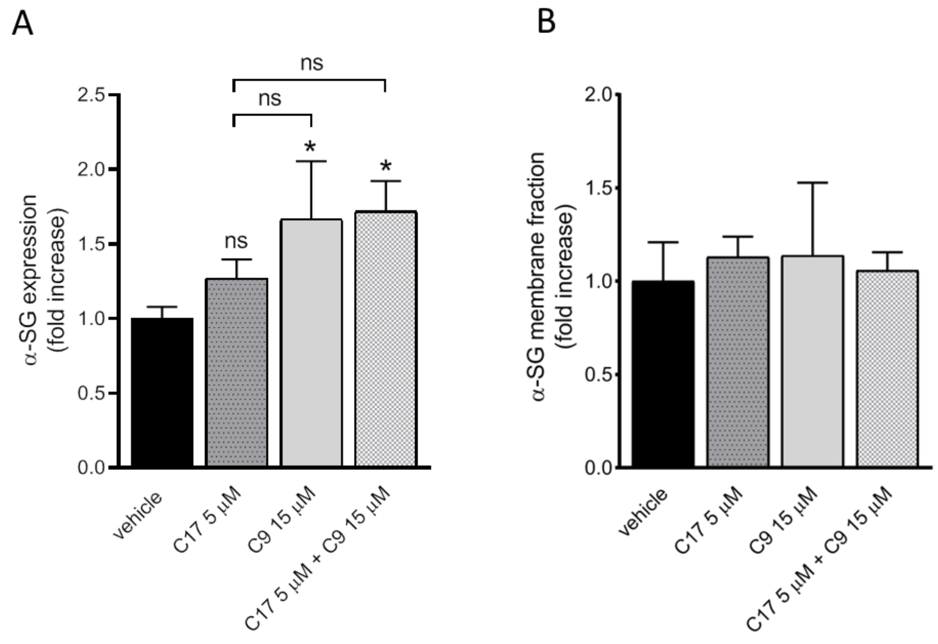

S3: the amount of α-SG accumulated by the incubation with C9 was not further increased by the co-administration of C17. Moreover, the C9-rescued protein did not localize at the sarcolemma.

Myogenic cells from a patient carrying the L31P/V247M α-SG mutations were differentiated for 7 days and treated for the last 72 h with either 1‰ DMSO (vehicle), C17, C9 or C17+C9 at the indicated concentrations. At the end of incubation, surface proteins were biotinylated. Then, myotubes were lysed and after quantification, proteins were pulled down by streptavidin-conjugated agarose beads. Recovered surface proteins and myotubes protein-lysates were analyzed by western blot. Graphs report the densitometric analysis from 4 independent experiments where **(A)** is the quantification of the total α-SG content and **(B)** the analysis of the α-SG membrane fraction. No additional increase in the content of α-SG was obtained by the co-administration of C9 and C17 in comparison to the administration of the sole C9. Moreover, the accumulated protein was unable to traffic and localize at the sarcolemma. Indeed, the surface protein analysis showed no difference among vehicle and CFTR corrector-treated samples. Statistical analysis was performed by One-way ANOVA test followed by multiple comparisons Tukey's test; n.s., $P > 0.05$; *, $P \leq 0.05$; **, $P \leq 0.01$; ***, $P \leq 0.001$.

S4

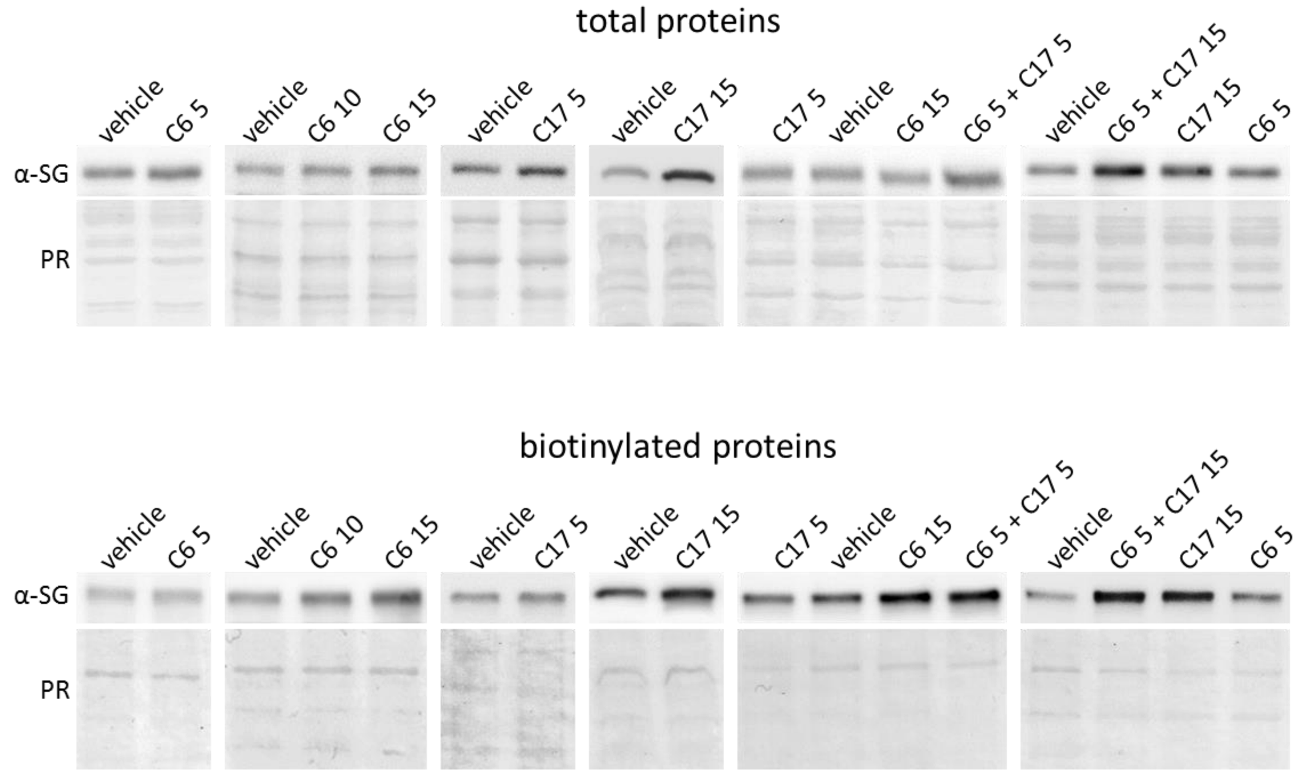

S4: Effects of C6 in combination with C17 (representative examples of Western blots).
Myogenic cells from a patient carrying the L31P/V247M α-SG mutations were differentiated for 7 days and treated for the last 72 h with either 1‰ DMSO (vehicle), C6, C17 or C6+C17 at the indicated concentrations (all μM). At the end of incubation, surface proteins were biotinylated. Then, myotubes were lysed and after quantification, 50 μg of proteins were subjected to pull down assay by streptavidin-conjugated agarose beads. Recovered surface proteins and 5 μg of myotubes protein lysates were analyzed by SDS-PAGE and western blot with antibodies against α-SG and the cytosolic protein β-actin used as loading control (western blot of input), Ponceau Red (PR) staining of the membranes was reported as control of protein loading.

S5

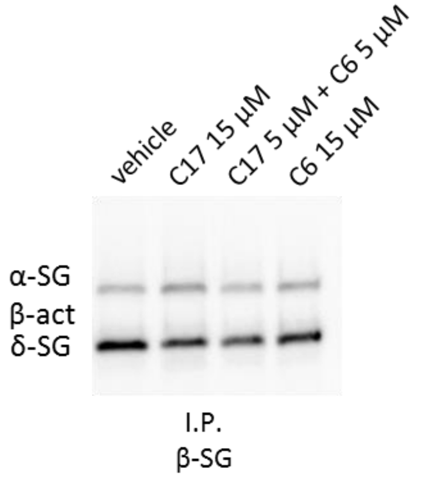

S5: α-SG mutant rescued by correctors C6 and C17 forms a functional SG-complex. Immunoprecipitation by the β-SG antibody.

Myogenic cells from a patient carrying the L31P/V247M α-SG mutations were differentiated for 7 days and treated for the last 72 hours with either 1‰ DMSO (vehicle), C17, C6, or C17+C6 at the indicated concentrations. At the end of incubation, myotubes were lysed with RIPA buffer without sodium deoxycholate to preserve the sarcoglycans' interactions. After quantification, 50 μg of proteins were subjected to immunoprecipitation with specific β-SG mouse monoclonal antibody. Immunocomplexes were resolved by SDS-PAGE and analyzed by western blot using the antibodies specific for α-SG, δ-SG and β-actin, used as control of specificity.

S6

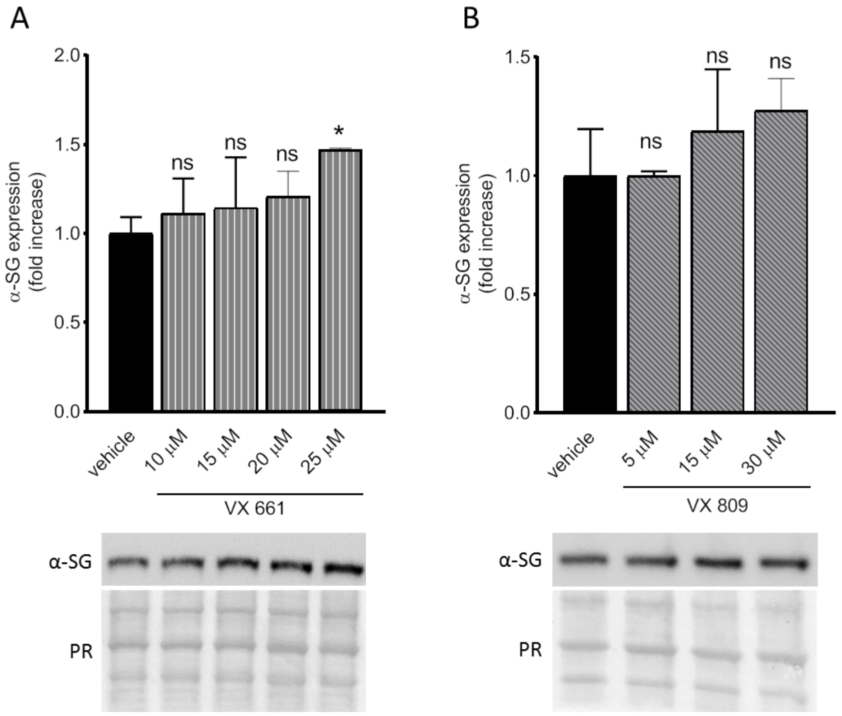

S6: identification of the working concentration of VX661 and VX809 CFTR correctors. VX661 and VX809 are correctors presently in use, in combination with the potentiator VX770, for the treatment of CF patients carrying the ΔF508 mutation in CFTR [24, 32]. In the frame of drugs repurposing, it could be valuable testing the efficacy of these two small molecules in rescuing sarcoglycan mutants. Therefore, we first identified the potential working concentration.

Myogenic cells from a patient carrying the L31P/V247M α-SG mutations were differentiated for 7 days and treated for the last 72 h with either 1‰ DMSO (vehicle), VX661 or VX809 at the indicated concentrations. At the end of incubation, myotubes were lysed and protein were analyzed by western blot. Quantification of α-SG content in myotube lysates was performed by densitometric analysis of 4 independent western blot experiments. Below the graphs showing the results of VX661 treatments **(A)** and of VX809 treatments **(B)**, are reported representative western blot examples; α-SG protein was revealed with a specific primary antibody Ponceau Red staining of the membranes was utilized to normalize the total amount of proteins loaded in each lane. Statistical analysis was performed by One-way ANOVA test followed by multiple comparisons Dunnett’s test; n.s., $P > 0.05$.

S7

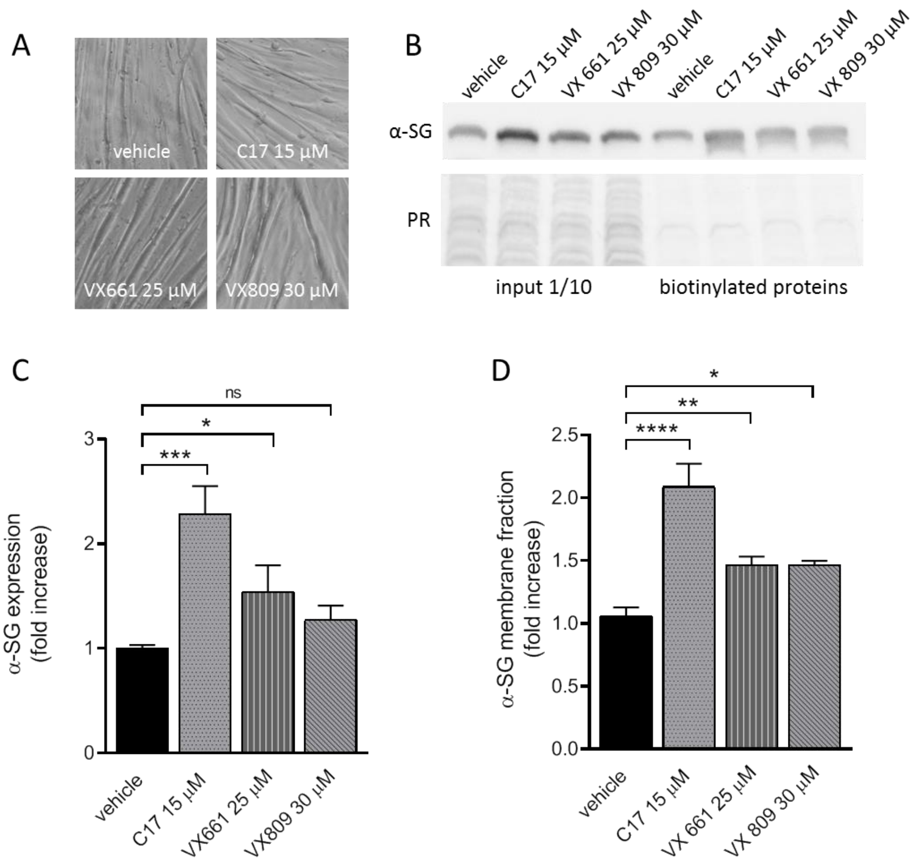

S7. Sarcolemma rescue of α-SG mutant by correctors VX661 or VX809 treatment. Myogenic cells from a patient carrying the L31P/V247M α-SG mutations were differentiated for 7 days and treated for the last 72 h with either 1‰ DMSO (vehicle), C17, VX661 or VX809 at the indicated concentrations. At the end of incubation, myotubes were treated as described in the legend of figure 2. (A) Phase contrast images of LGMD2D myotubes recorded at the end of treatments showing no evident toxic effect. (B) Representative western blot of total protein lysates (input) (left part) and of the biotinylated membrane fraction (right part) probed with the antibody specific for α-SG. Ponceau Red (PR) staining was reported to estimate protein loading. (C) Quantification of total α-SG content and (D) quantification of α-SG level at the sarcolemma by densitometric analysis of 3 independent experiments. Statistical analysis was performed by One-way ANOVA test followed by multiple comparisons Tukey's test; ns, $P > 0.05$; *, $P \leq 0.05$; **, $P \leq 0.01$; ***, $P \leq 0.001$; ****, $P \leq 0.0001$. While C17 induced a robust increment of the α-SG level, VX661 and VX809 produced a modest 1.5 fold in comparison to the untreated cells, and this variation was statistically significant for VX661 only. Nevertheless, when the membrane localization is analyzed a similar extent of α-SG was delivered to the cell surface by both VX661 and VX809, even if less pronounced that what achievable by C17 treatment.

S8

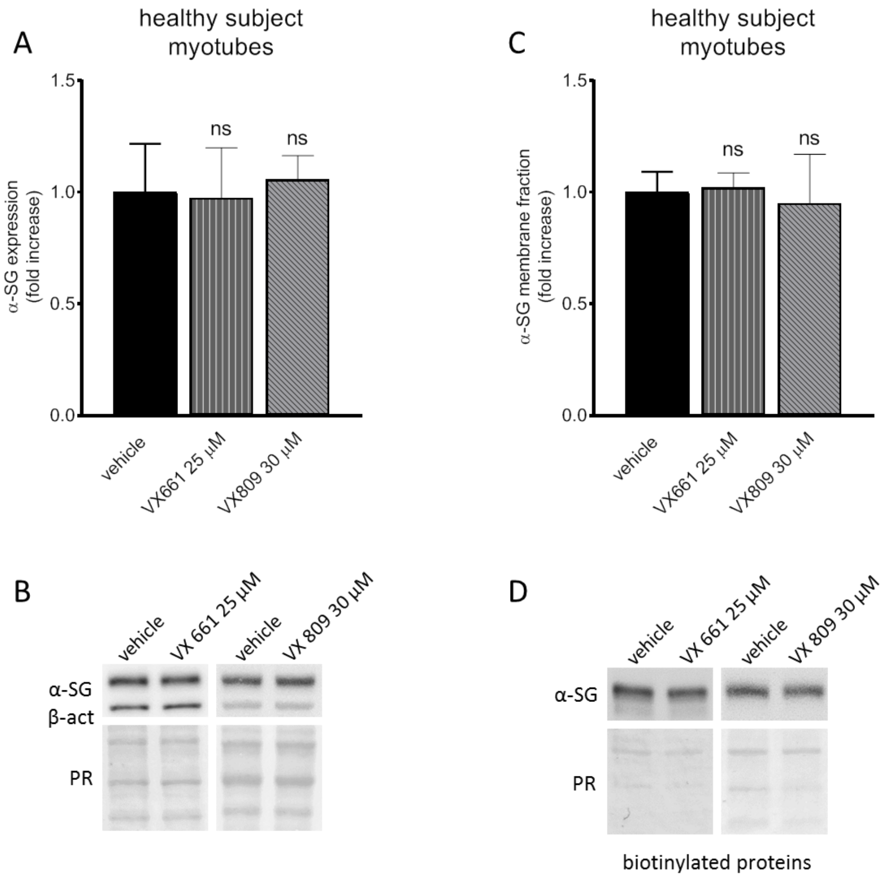

S8: VX661 and VX809 are ineffective in healthy subject's myotubes. Myogenic cells from a healthy subject were differentiated for 7 days and treated for the last 72 h with either 1‰ DMSO (vehicle), VX661 25 µM or VX809 30µM. At the end of incubation, myotubes were lysed and after quantification, proteins were pulled down by streptavidin-conjugated agarose beads. Surface protein were subsequently analysed by western blot. **(A)** Quantification of α-SG content in myotube lysates performed by densitometric analysis of 4 independent western blot experiments and **(B)** representative western blots, α-SG protein was revealed with a specific primary antibody; β-actin staining was utilized to normalize the total amount of proteins loaded in each lane. No statistical difference was observed among CFTR correctors and vehicle treated samples. **(C)** Quantification of the sarcolemma fraction of α-SG performed by densitometric analysis of 4 independent western blot experiments and **(D)** representative examples, α-SG protein was revealed with a specific primary antibody; Ponceau Red is reported to estimate protein loading. No statistical difference was observed in the content of the α-SG present at the sarcolemma among the different samples. Statistical analysis was performed by One-way ANOVA.

S9

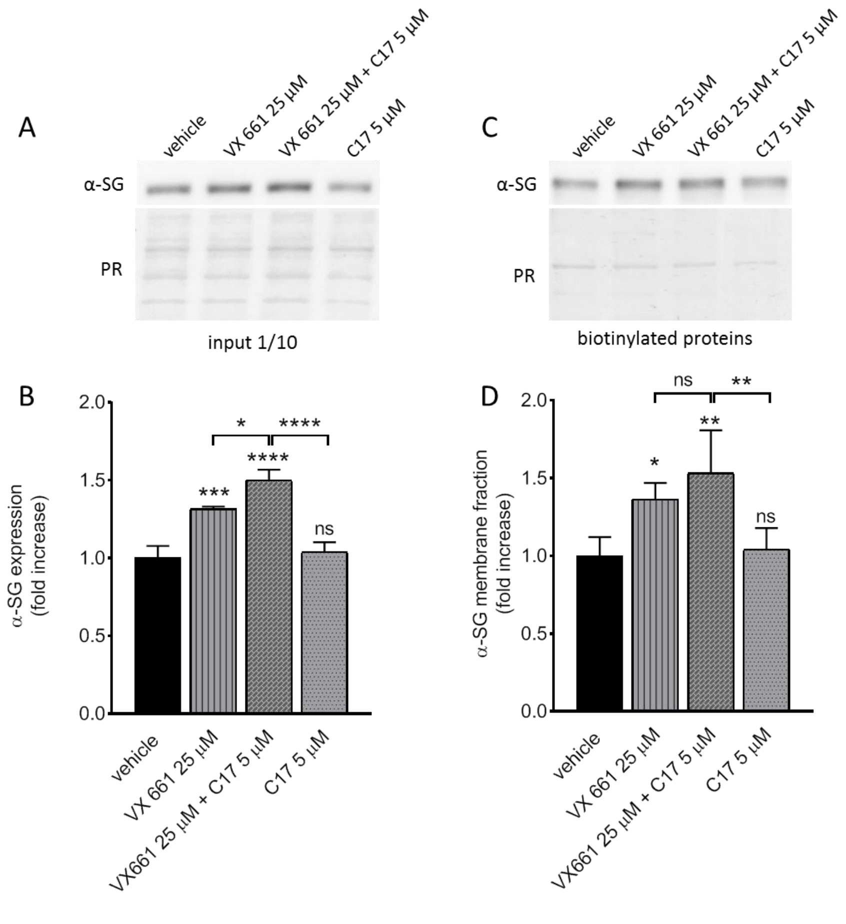

S9. Additive effect of VX661 in combination with C17. Myogenic cells from a patient carrying the L31P/V247M α-SG mutations were differentiated for 7 days and treated for the last 78 h with either 1‰ DMSO (vehicle), VX661, C17 or VX661+C17 at the indicated concentrations. At the end of incubation, myotubes were treated as described in the legend of Figure 2. (A) Representative western blot of total protein lysates (input) probed with the antibody specific for α-SG, Ponceau Red (PR) staining was reported for protein normalization. (B) Quantification of global α-SG content by densitometric analysis. (C) Representative western blot of the biotinylated membrane fraction probed with the antibody specific for α-SG, Ponceau Red (PR) staining was reported to estimate protein loading. (D) Quantification of α-SG membrane fraction by densitometric analysis. Densitometric analyses were from 3 independent experiments. Statistical analysis was performed by One-way ANOVA test followed by multiple comparisons Tukey's test; ns, $P > 0.05$; *, $P \leq 0.05$; **, $P \leq 0.01$; ***, $P \leq 0.001$; ****, $P \leq 0.0001$. When an ineffective dose of C17 is co-administered with VX661, it is possible to observe an extra accumulation of the mutant in comparison to the VX661 single administration. This effect is also observable at the sarcolemma, where an higher amount of α-SG localized upon the dual treatment. Therefore, it possible to suppose that also VX661 acts in additive way with C17 in rescuing sarcoglycan mutants.

S10

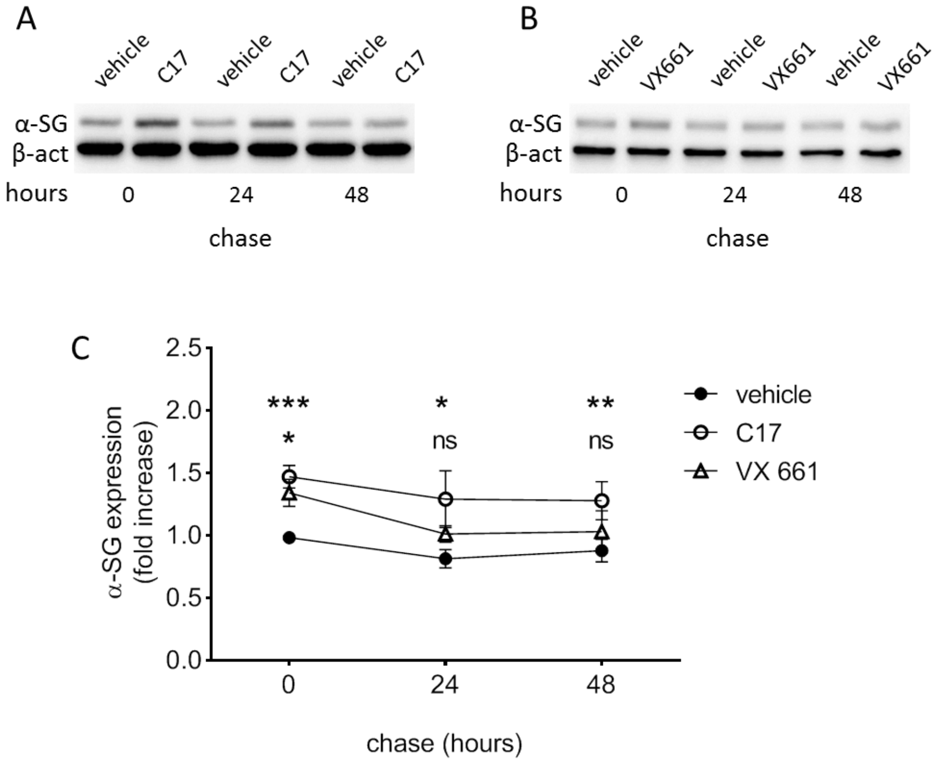

S10: Evaluation of the duration of CFTR corrector effect. Myogenic cells from a patient carrying the L31P/V247M α-SG mutations were differentiated for 7 days and treated for the last 78 hours with either 1‰ DMSO (vehicle), C17 15 µM or VX661 25 µM. At the end of treatment corrector containing medium was removed and replaced with fresh medium. Myotubes were then lysed at 0, 24 and 48 hours after medium withdrawal. The amount of a-SG at each time point was quantified by densitometric analysis of at least 4 independent western blot experiments. **(A)** representative western blot of proteins form myotubes pre-treated with VX661; **(B)** representative western blot of proteins form myotubes pre-treated with VX809; **(C)** graph showing the densitometric quantification of α-SG. Each value is the mean (+/-) SD and is referred to the a-SG content measured in myotube treated with vehicle at time 0. Statistical analysis was performed by One-way ANOVA test followed by multiple comparisons Tukey's test; n.s., $P > 0.05$; *, $P \leq 0.05$; **, $P \leq 0.01$; ***, $P \leq 0.001$.

S11

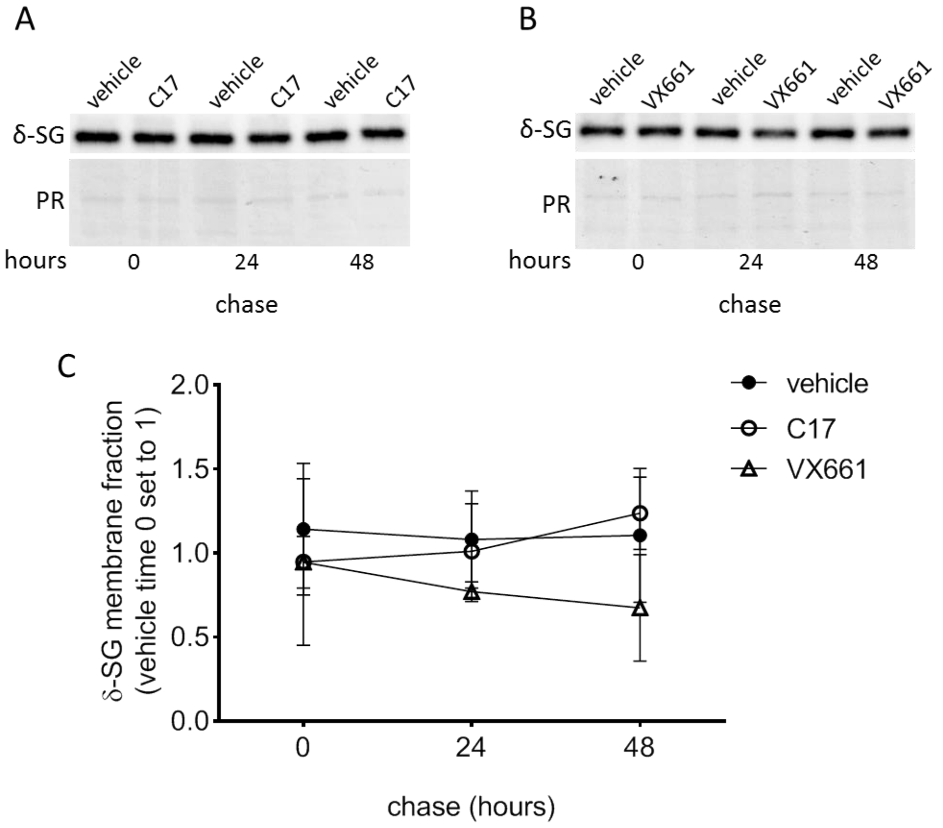

S11: The endogenous δ-SG protein is stable at the sarcolemma

Myogenic cells from a patient carrying the L31P/V247M α-SG mutations were differentiated for 7 days and treated for the last 78 hours with either 1‰ DMSO (vehicle), C17 15 µM or VX661 25 µM. At the end of treatment corrector containing medium was removed and replaced with fresh medium. Myotubes were then treated and lysed, at the indicated time points, as described in the legend of Figure 2. Representative western blots of the biotinylated membrane fraction from myotubes treated with vehicle or C17 **(A)**; with vehicle or VX661 **(B)** probed with antibodies specific for δ-SG; Ponceau Red (PR) staining of membranes is reported to estimate protein loading. The content of δ-SG resident in the sarcolemma of cells pre-treated with either vehicle, C17 or VX661 **(C)** was quantified by densitometric analysis of 4 independent experiments. Values are referred to the amount of δ-SG present in vehicle-treated cells at time 0. No difference in δ-SG content at the sarcolemma can be appreciated among vehicle- and C17 pre-treated samples during time. Conversely, a slow reduction seems to appear in sample pre-treated with VX661, however values were not statistical significant. Statistical analysis was performed by One-way ANOVA test.
